# A novel risk stratification model for STEMI after primary PCI: global longitudinal strain and deep neural network assisted myocardial contrast echocardiography quantitative analysis

**DOI:** 10.3389/fcvm.2023.1140025

**Published:** 2023-04-27

**Authors:** Mingqi Li, Dewen Zeng, Yanxiang Zhou, Jinling Chen, Sheng Cao, Hongning Song, Bo Hu, Wenyue Yuan, Jing Chen, Yuanting Yang, Hao Wang, Hongwen Fei, Yiyu Shi, Qing Zhou

**Affiliations:** ^1^ Department of Ultrasound Imaging, Renmin Hospital of Wuhan University, Wuhan, China; ^2^ Department of Computer Science and Engineering, University of Notre Dame, South Bend, IN, United States; ^3^ Department of Cardiology, Renmin Hospital of Wuhan University, Wuhan, China; ^4^ Department of Cardiology, Guangdong Cardiovascular Institute, Guangdong Provincial People's Hospital (Guangdong Academy of Medical Sciences), Southern Medical University, Guangzhou, China

**Keywords:** ST-segment elevation myocardial infarction, deep neural network, myocardial contrast echocardiography, microvascular perfusion, prognosis

## Abstract

**Background:**

In ST-segment elevation myocardial infarction (STEMI) with the restoration of TIMI 3 flow by percutaneous coronary intervention (PCI), visually defined microvascular obstruction (MVO) was shown to be the predictor of poor prognosis, but not an ideal risk stratification method. We intend to introduce deep neural network (DNN) assisted myocardial contrast echocardiography (MCE) quantitative analysis and propose a better risk stratification model.

**Methods:**

194 STEMI patients with successful primary PCI with at least 6 months follow-up were included. MCE was performed within 48 h after PCI. The major adverse cardiovascular events (MACE) were defined as cardiac death, congestive heart failure, reinfarction, stroke, and recurrent angina. The perfusion parameters were derived from a DNN-based myocardial segmentation framework. Three patterns of visual microvascular perfusion (MVP) qualitative analysis: normal, delay, and MVO. Clinical markers and imaging features, including global longitudinal strain (GLS) were analyzed. A calculator for risk was constructed and validated with bootstrap resampling.

**Results:**

The time-cost for processing 7,403 MCE frames is 773 s. The correlation coefficients of microvascular blood flow (MBF) were 0.99 to 0.97 for intra-observer and inter-observer variability. 38 patients met MACE in 6-month follow-up. We proposed A risk prediction model based on MBF [HR: 0.93 (0.91–0.95)] in culprit lesion areas and GLS [HR: 0.80 (0.73–0.88)]. At the best risk threshold of 40%, the AUC was 0.95 (sensitivity: 0.84, specificity: 0.94), better than visual MVP method (AUC: 0.70, Sensitivity: 0.89, Specificity: 0.40, IDI: −0.49). The Kaplan-Meier curves showed that the proposed risk prediction model allowed for better risk stratification.

**Conclusion:**

The MBF + GLS model allowed more accurate risk stratification of STEMI after PCI than visual qualitative analysis. The DNN-assisted MCE quantitative analysis is an objective, efficient and reproducible method to evaluate microvascular perfusion.

## 1. Introduction

Many studies revealed a significant prevalence of microvascular obstruction (MVO) after successful revascularization in ST-elevation myocardial infarction (STEMI) patients (1–3). And coronary microvascular dysfunction detected by angiography, cardiac magnetic resonance, or myocardial contrast echocardiography (MCE) visual qualitative analysis is associated with adverse outcomes (4–6).

MCE allows for the non-invasive and cost-effective assessment of microvascular perfusion by qualitative and quantitative analysis. The previous study proved that delayed microvascular perfusion (dMVP) and MVO evaluated by MCE qualitative analysis after revascularization were independent predictors of adverse events (4). Although qualitative analysis achieved risk stratification, only 27% of MVO patients had adverse events at 6-month. It indicates an overestimation of the short-term prognosis of patients by MVO. Furthermore, it is a subjective and highly experience-dependent diagnosis based on human visual impressions. The quantitative analysis could provide more objective information than qualitative analysis. It appears to have additional value over visual analysis in detecting myocardial blood flow abnormalities (7). Nevertheless, MCE quantitative analysis is complex and time-consuming and has limited repeatability by current commercial software. Evidence for the prognostic value of MCE quantitative analysis in STEMI patients undergoing percutaneous coronary intervention (PCI) is lacking. Whether accurate quantitative analysis improves prognostic predictive value and how to use it for risk stratification is a pressing clinical question.

Our research team previously proposed a deep neural network (DNN) for MCE quantitative analysis, which can automatically trace and segment the myocardium in three apical chamber views and output perfusion parameters for each segment (8).

Therefore, we intend to use DNN-assisted MCE quantitative analysis and propose a risk probability prediction model for STEMI after successful PCI.

## 2. Methods

### 2.1. Study population

**Figure 1:**
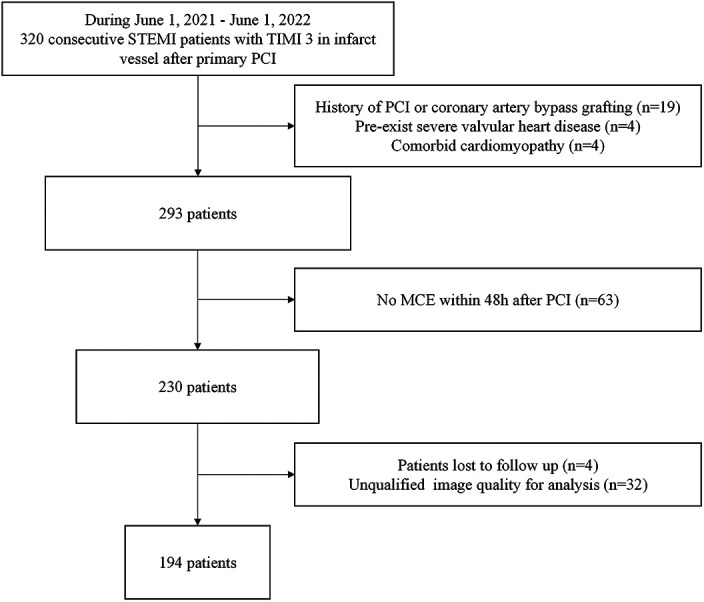
Flow chart depicting patient inclusion and exclusion criteria. MCE, myocardial contrast echocardiography; PCI, percutaneous coronary intervention; STEMI, ST-elevation myocardial infarction.

We performed a single center, prospective analysis of retrospectively acquired MCE studies. The process for importing data is shown in Figure 1. The inclusion criteria: consecutive STEMI patients with TIMI 3 in infarct vessel after primary PCI from June 1, 2021, to June 1, 2022. Exclusion criteria: (1) History of PCI or coronary artery bypass grafting; (2) Pre-exist severe valvular disease; (3) Comorbid cardiomyopathy; (4) No MCE within 48 h after PCI. (5) Patients lost to follow up; (6) Unqualified image quality for analysis. Finally, 194 patients were included in the study. Medication administration during hospitalization and after discharge involves antianginal, antithrombotic, *β*-blocker, ACE inhibitor/ARB, and lipid-lowering medication.

The single culprit vessel was identified and revascularized based on the (leads with ST-segment elevation and Q waves), routine echocardiography (corresponding coronary artery with abnormal segmental wall motion), and coronary angiography (acute thrombotic total or subtotal occlusion) (9, 10). Decision-making related to the PCI strategy depended on the individual physicians according to the guideline (11).

### 2.2. Outcome assessment

Patients in this study were either seen for regular monthly follow-up visits at the hospital or received telephone follow-up from a physician if they were unable to come to the hospital. Follow-up information was obtained from medical records. All patients were followed up for at least 6 months (medium: 333 days, Q1–Q3: 207–432 days) unless met the endpoint. The endpoint of this study was composite major adverse cardiac events (MACE) defined as cardiac death, hospitalization for congestive heart failure, reinfarction, stroke, and recurrent angina. Reinfarction was defined as the recurrent elevation of cardiac enzymes with recurrent chest pain and new ST-segment elevation. Recurrent angina was defined as ischemic chest pain with either new ST-segment or T wave changes at rest or on exercise testing.

### 2.3. Routine and contrast echocardiography

Echocardiography was performed within 48 h after PCI on commercially available ultrasound systems with a contrast-specific multipulse amplitude modulation imaging algorithm (Philips 7C, Philips Medical Systems, Best, Netherlands) equipped with a broadband transducer S5–1.

Routine echocardiographic images were obtained from apical two-chamber (A2C), three-chamber (A3C), and four-chamber (A4C) views with 3 cardiac cycles. Global longitudinal strain (GLS) was automatically generated by “Automatic Strain LV” module of Qlab 13 (Philips). Left atrial volume (LAV) was measured using the bi-plane Simpson method. The peak mitral valve velocity of early € and late (*A*) diastole, the myocardial peak early velocity at medial mitral annulus (*e*′) and tricuspid annular plane systolic excursion (TAPSE) were obtained according to the guidelines of the American Society of Echocardiography.

SonoVue (Bracco Research SA, Geneva, Switzerland) powder as ultrasound enhancing agent (UEA) was dissolved to 5 ml of liquid. Then 2.5 ml of it was diluted to 15 ml with normal saline. The intravenous continuous infusion rate (mean as 2.5–3 ml/min) was adjusted to obtain maximal opacification of the myocardium with minimal attenuation throughout the examination. Real-time contrast imaging at a low mechanical index setting (0.15–0.19) and frame rates of 20–30 Hz was utilized with transient high mechanical index (1.20–1.30) flash (10–15 frames) was used to clear myocardial microbubbles. Before UEA infusion, time gain compensation, gain, and compression settings were adjusted to reduce background signals from the myocardium or blood. After UEA infusion, once the attenuation was minimized and apical swirling was not present, A2C, A3C, and A4C views were digitally captured with 13 to 15 cardiac cycles. All settings remained unchanged throughout the study.

Left ventricular end-diastolic volume (LVEDV) and LVEF were measured using the bi-plane Simpson method. Wall motion score index (WMSI) was calculated as the average score of 17 segments according to the scoring systems as follows (3): wall motion: 1 = normal, 2 = hypokinesis, 3 = akinesis, and 4 = dyskinesis; Microvascular perfusion (MVP) (4): 1 = normal (time of replenishment completion: <4 s in all segments supplied by infarct related artery), 2 = delayed MVP (time of replenishment completion: 4s-8s was observed > 1 segment of infarct zone), 3 = MVO (persistent perfusion defect was observed > 1 segment of infarct zone). Microvascular perfusion score index (MPSI) was calculated as the average score of the 17 segments.

### 2.4. Acquisition of perfusion parameters by deep neural network

**Figure 2:**
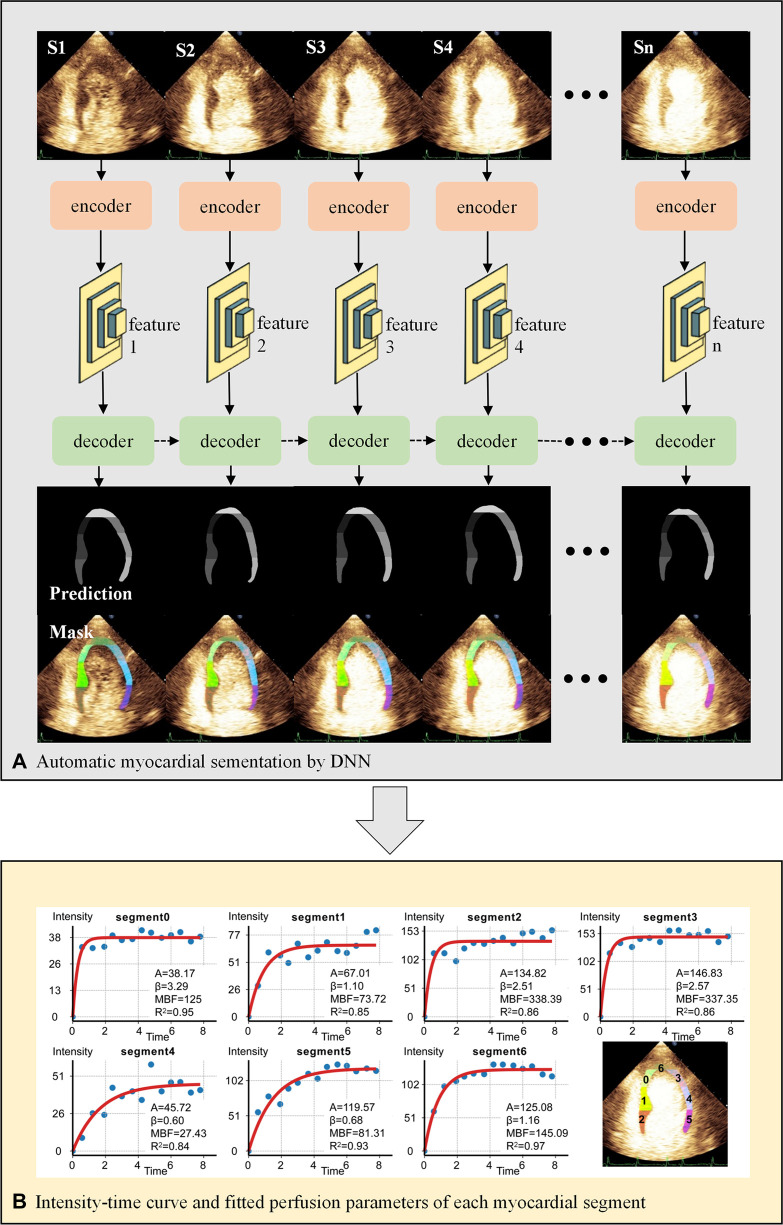
Workflow of MCE quantitative analysis. (**A**) The U-net-based encoder extracts the image features 1-n of each end-systolic frame, which are then put into the decoder for myocardial segmentation. The decoder consists of hierarchical ConvLSTMs and can incorporate temporal information between MCE frames. The dashed arrows represent the temporal information of the myocardium in the previous frame as additional features to enhance the segmentation of the current frame. (**B**) Automatic calculation of perfusion parameters for each myocardial segment by fitting equation. DNN, deep neural network; S1-n, end-systolic frames of consecutive cardiac cycles.

Figure 2 presents the workflow of MCE quantitative analysis.

#### 2.4.1. Deep neural network

We proposed a DNN framework with temporal consistency for automatic MCE myocardial segmentation in the previous study (8). The encoder is designed based on U-net, extracting five features from the up-sampling layers. The decoder contains hierarchical convolutional LSTMs for myocardial segmentation. The DNN was trained and validated internally by the MCE dataset of Guangdong Provincial People's Hospital.

#### 2.4.2. Acquisition of perfusion parameters of each segment

First, the DICOM file was decomposed into a sequence of images. We removed the text information of images by automated image cropping process, except for the acoustic window. Then end-systolic frames after “flash” at the T wave were selected to be analyzed. DNN performed automatic myocardial segmentation and labeled 7 segments on each frame as the regions of interest (ROIs).

An experienced echocardiographer verified the ROIs and classified them into “good segmentation” and “need for correction”. The echocardiographer modified ROIs to ensure that segments were correctly marked.

Finally, the time-intensity replenishment curves were generated by the following exponential equation $Y=A\times (1-e^{-\beta t})$, which were recommended by the UEA guideline (7). *Y* is the intensity at time *t*, *A* is the plateau microvascular contrast intensity, and β depicts mean micro-bubble velocity. The product of *A* and β represents microvascular blood flow (MBF). The intensity unit was the gray-scale value ranging from 0 to 255.

#### 2.4.3. Definition of culprit-perfusion parameters

As shown in Supplementary Figure S1, the myocardium was divided into 17 segments ascribed to coronary territories. Culprit-perfusion parameters were calculated as the arithmetic mean of the segments supplied by the single infarct-related artery, which was defined and revascularized during the PCI based on the coronary angiogram findings and electrocardiogram (12). (e.g., assuming the culprit vessel is RCA, then culprit-MBF is the arithmetic mean of MBF of the inferior wall and middle and basal segments of the inferior septum).

#### 2.4.4. Measurement reproducibility

Since the parameters of the trained neural network are fixed, the myocardial segmentation framework works with 100 percent reproducibility in the same sample. The variability of perfusion parameter measurement was only caused by the manual correction, which was assessed in 25 randomly selected patients. The intra-observer variability was assessed two months apart by one echocardiographer. The inter-observer variability was assessed by two independent echocardiographers who were asked to review the DNN-segmented myocardial ROI and make corrections if necessary.

### 2.5. Statistical analysis

Data were analyzed using R (http://www.R-project.org), Empower Stats software (http://www.empower.stats.com, X&Y solutions, Inc., Boston, MA, USA). Continuous variables were analyzed using the t-test (normal distribution) or Kruskal–Wallis rank-sum test (nonnormal distribution), and categorical variables were analyzed using the *χ*^2^ test. Intra-observer variability was assessed using intraclass correlation coefficients. Variables screening and prognostic prediction model construction were performed by the following steps: (1) LASSO regression (lambda.1se method); The selected variables plus other clinical and echocardiographic variables based on clinical reasoning and literature review were subjected to (2) near zero variance check; (3) collinearity check (variance inflation factor stepwise selection); (4) recursive feature elimination. Finally, the *β* coefficients of the selected variables by Cox regression analysis fit into the risk prediction equation (13). The performance of the proposed model was assessed by discrimination, calibration, and clinical utility. The discriminative ability was determined by the area under the receiver operating characteristic curve (ROC), which ranged from 0.5 to 1. The calibration was performed by a visual calibration plot comparing the predicted and actual probability of MACE. Clinical utility was measured using decision curve analysis, where the standardized net benefit (sNB) was calculated by (14):

$$\mathrm{sNB}=\mathrm{TPR}-\frac{\mathrm{Risk}\;\mathrm{Threshold}}{1-\mathrm{Risk}\;\mathrm{Threshold}}\times \frac{1-\mathrm{Prevalence}}{\mathrm{Prevalence}}\times \mathrm{FPR}$$

TPR, true positive rate; FPR, false positive rate. The proposed model was subjected to 500 bootstrap resampling for internal validation. Kaplan–Meier curves were plotted to depict event-free survival rate over time. The integrated discrimination improvement (IDI) was used for evaluating the incremental value of the proposed model relative to other models. Two-sided *P* values with *P *< 0.05 were considered statistically significant.

## 3. RESULT

### 3.1. Baseline characteristics

All patients were followed up for at least 6 months (medium, Q1–Q3: 333 days, 207–432 days), unless met the endpoint. 38/45 patients met the MACE in 6-month follow-up. Patients' baseline characteristics are summarized in Table 1. There were higher Killip level, admission glucose, NT-proBNP, Hs-CRP, creatinine, cTnI-Ultra, WMSI, MPSI in patients with MACE during follow-up. There was a higher proportion of dMVP and MVO in patients with MACE (dMVP: 33.33% vs. 11.11%; MVO: 55.55% vs. 11.11%, both *P *< 0.01). And the left ventricular ejection fraction (LVEF), GLS and culprit-MBF were lower in patients with MACE (GLS: −11.23% ± 3.60% vs. −15.28% ± 3.87%; culprit-MBF: 30.62 ± 14.20 vs. 74.07 ± 37.22, both *P *< 0.01).

**Table 1:** Distribution of baseline characteristics among clinical outcomes.

	MACE	Event-free	*P*-value
*N*	45	149	
Age (years)	63 ± 13	61 ± 11	0.19
Male	34 (75.56%)	124 (83.22%)	0.25
Time window to PCI	8.00 (4.00–17.00)	6.00 (3.00–12.00)	0.17
**Killip**			**<0.01**
I	10 (22.22%)	75 (50.34%)	
II	8 (17.78%)	35 (23.49%)	
III	10 (22.22%)	14 (9.40%)	
IV	17 (37.78%)	25 (16.78%)	
Hypertension	31 (68.89%)	87 (58.39%)	0.21
Diabetes mellitus	16 (35.56%)	42 (28.19%)	0.34
Systolic blood pressure (mmHg)	122.09 ± 18.68	124.87 ± 16.14	0.42
Admission glucose (mmol/L)	8.75 ± 3.10	7.65 ± 2.81	**0**.**03**
NT-proBNP (ng/ml)	2,130 (805–4,927)	712 (264–1,526)	**<0.01**
Leukocyte (10^9^/L)	11.71 ± 3.62	10.91 ± 3.28	0.17
Hs-CRP (mg/L)	4.48 (1.37–8.98)	1.49 (0.50–5.13)	**0**.**02**
Creatinine (umol/L)	84.49 ± 35.81	72.68 ± 22.64	**<0.01**
CK-MB (ng/ml)	112.94 (47.67–256.23)	87.12 (37.26–189.00)	0.09
Myoglobin (ug/L)	1,000.00 (392.33–1,000.00)	918.28 (191.63–1,000.00)	0.11
cTnI-Ultra (ng/ml)	43.81 ± 12.01	35.10 ± 18.05	**<0.01**
**Number of stenosed vessels**			0.75
1	27 (60.00%)	98 (65.77%)	
2	15 (33.33%)	41 (27.52%)	
3	3 (6.67%)	10 (6.71%)	
**Culprit vessel**			0.25
LAD	27 (60.00%)	75 (50.34%)	
LCx	9 (20.00%)	25 (16.78%)	
RCA	9 (20.00%)	49 (32.89%)	
LVEF (%)	44.65 ± 8.73	52.29 ± 8.30	**< 0.01**
LVEDV (ml)	127.13 ± 38.47	119.65 ± 31.87	0.19
LAV Index (ml/m^2^)	28.43 ± 10.31	26.52 ± 9.99	0.27
*E*/*A*	0.79 ± 0.50	0.81 ± 0.31	0.73
*E*/*e*′	12.09 ± 3.82	11.30 ± 3.56	0.20
TAPSE (mm)	19.60 ± 2.61	19.63 ± 2.25	0.94
WMSI	1.52 ± 0.38	1.23 ± 0.26	**<0.01**
**MVP**			**<0.01**
Normal MVP	5 (11.11%)	61 (40.94%)	
Delayed MVP	15 (33.33%)	47 (31.54%)	
MVO	25 (55.55%)	41 (27.52%)	
MPSI	1.36 ± 0.26	1.17 ± 0.20	**<0.01**
GLS (%)	−11.23 ± 3.60	−15.28 ± 3.87	**<0.01**
Culprit-*A* (IU)	62.64 ± 23.99	82.26 ± 24.37	**<0.01**
Culprit-*β* (s^−1^)	0.54 ± 0.25	1.11 ± 0.85	**<0.01**
Culprit-MBF (IU/s)	30.62 ± 14.20	74.07 ± 37.22	**<0.01**

Data are presented as mean ± standard deviation, *n* (%), or media (Q1–Q3).

CK-MB, creatine kinase myocardial band; cTnI, cardiac troponin I; *E*/*A*, The ratio of peak mitral valve velocity of early (*E*) and late (*A*) diastole. *E*/*e*′, The ratio of *E* and myocardial peak early velocity at medial mitral annulus. GLS, global longitudinal strain; Hs-CRP, high-sensitivity C-reactive protein; IU, intensity unit; LAV-index, Left atrial volume divided by body surface area; LVEDV, left ventricular end-diastolic volume; LVEF, left ventricular ejection fraction; MBF, microvascular blood flow; MPSI, myocardial perfusion score index; MVO, microvascular obstruction; MVP, microvascular perfusion; NT-proBNP, N-terminal pro b-type natriuretic peptide; PCI, percutaneous coronary intervention; TAPSE, tricuspid annular plane systolic excursion; WMSI, wall motion score index.

### 3.2. Risk prediction model derivation and internal validation

For model derivation, we performed the following steps to filter the predictors: After LASSO regression (see Supplementary Figure S2 for detail), five potential predictors were selected (Creatinine, LVEF, WMSI, GLS, Culprit-MBF). These variables plus other clinical and echocardiographic variables (age, Killip level, time window to PCI, admission glucose, NT-proBNP, CK-MB, Myoglobin, cTnI-Ultra, MPSI and MVP) based on clinical reasoning and literature review were subjected to near zero variance check, collinearity check. Finally, the culprit-MBF and GLS were selected by recursive feature elimination. Multivariate Cox regression analysis showed that the HR of culprit-MBF and GLS were 0.93 (95% CI: 0.91–0.95) and 0.80 (95% CI: 0.73–0.88). After 100-times bootstrap for slope shrinkage (*β* coefficients × 0.9,437). The final model was:

$$\mathrm{Predicted}\;\mathrm{risk}=1-S_{0}^{\mathrm{EXP}[-0.06872\times \mathrm{Culprit}\;\mathrm{MBF}-0.20747\times \mathrm{GLS}+7.75130]}$$

We provided a calculator for 1-, 3-, 6-, 9- and 12-month risk prediction in Supplementary Excel for ease of use. The baseline survival rate $S_{0}$ could be queried from sheet 2 of Supplementary Excel. The risk probability of MACE is automatically output by entering the culprit-MBF and GLS.

**Figure 3:**
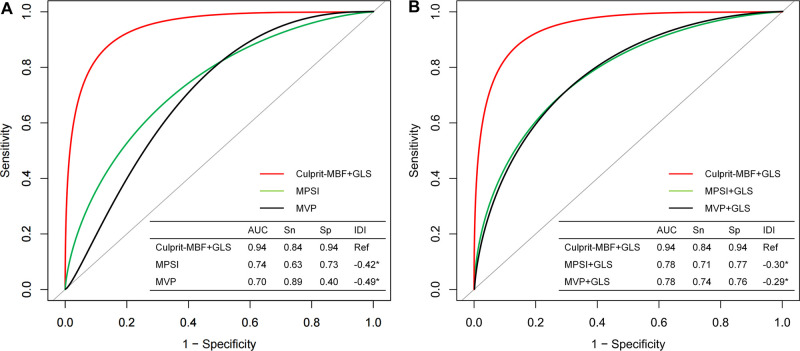
The discrimination of different models for MACE at 6-month follow-up. (**A**) Comparison of the proposed model with traditional visual qualitative analysis. (**B**) Comparison of the proposed model with the qualitative analysis + GLS model. MBF, microvascular blood flow; MPSI, myocardial perfusion score index; MVP, microvascular perfusion; Sn, sensitivity; Sp, specificity; IDI, Integrated Discrimination Improvement; *, *P* value < 0.05.

**Figure 4:**
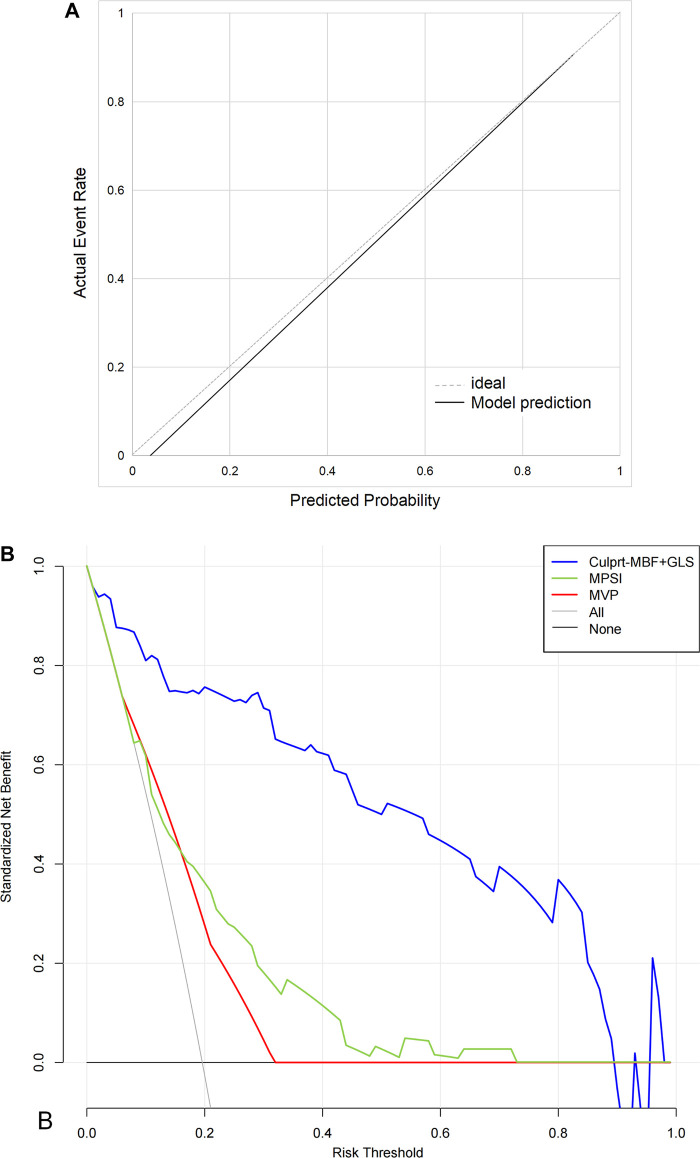
The calibration curves and decision curve. (**A**) The calibration curves. Perfect prediction would correspond to the 45° dashed line. The black line represents the observed model performance. (**B**) The decision curve. “None” model means that all patients are considered as not having MACE. “All” model means that all patients are considered as having MACE. The preferred model is considered to have the highest standardized net benefit for a given threshold range.

The C-index of the proposed model in internal validation by bootstrapping was 0.90 (0.86–0.93). Prognostic prediction performance at 1-, 3-, 6-, 9- and 12-month follow-up was shown in Supplementary Table S2, respectively. Table 2 compares the discrimination of the various models in terms of 6-month prognosis prediction, demonstrating the best discrimination of the Culprit-MBF + GLS model at a threshold risk of 40% (AUC: 0.94, Sensitivity: 0.84, Specificity: 0.94). Figure 3 compares the ROC of Culprit-MBF + GLS, MVP, MPSI, MVP + GLS, and MPSI + GLS. The IDI of the proposed model is 0.49, 0.42, 0.29, and 0.30 (all *P *< 0.01), respectively. The discrimination of the proposed model at different risk threshold settings is available in Table 3. A calibration curve of proposed model is presented in Figure 4A, which shows that the predicted risks of MACE by the model agreed well with the actual event rates. The clinical decision curve (Figure 4B) illustrated that within a wide range of threshold probabilities, clinical decision making by proposed model provided a greater sNB than by MVP or MPSI. For example, using 30% of risk as the clinical intervention threshold, 71% of patients with MACE received treatment without false positive patients by culprit-MBF + GLS model, 53% and 66% more than MPSI and MVP model, respectively.

**Table 2:** Comparison of the discrimination of different models predicting MACE at 6-month.

	IDI	*P*-value	AUC	Threshold	Sn	Sp	C-index
Predicted risk*	Ref	Ref	0.94	40%	0.84	0.94	0.90 (0.86–0.93)
MVP	−0.49	<0.01	0.70	dMVP	0.89	0.40	0.67 (0.60–0.74)
	–	–	–	MVO	0.55	0.71	–
LVEF	−0.41	<0.01	0.74	41.65%	0.45	0.93	0.71 (0.64–0.79)
MPSI	−0.42	<0.01	0.74	1.2,647	0.63	0.73	0.70 (0.62–0.78)
WMSI	−0.34	<0.01	0.75	1.3,824	0.61	0.84	0.71 (0.63–0.79)
GLS	−0.32	<0.01	0.77	−12.06%	0.71	0.76	0.75 (0.68–0.83)
MPSI + GLS	−0.30	<0.01	0.78	−1.1,694	0.71	0.77	0.76 (0.68–0.83)
MVP + GLS	−0.29	<0.01	0.78	−1.1,166	0.74	0.76	0.76 (0.69–0.83)
Culprit-MBF	−0.10	0.05	0.89	49.10 IU/s	0.94	0.68	0.85 (0.81–0.89)

GLS, global longitudinal strain; LVEF, left ventricular ejection fraction; MBF, microvascular blood flow; MPSI, myocardial perfusion score index; MVO, microvascular obstruction; MVP, microvascular perfusion; AUC, Area Under the Curve; Sn, sensitivity; Sp, specificity; IDI, integrated discrimination improvement.

^*^ Calculated by the proposed model of Culprit-MBF + GLS.

**Table 3:** The discrimination of the proposed model at different risk threshold settings.

Threshold risk	Sensitivity	Specificity	Accuracy	PPV	NPV
10%	1.00	0.62	0.69	0.39	1
20%	0.92	0.79	0.82	0.52	0.97
30%	0.84	0.88	0.87	0.63	0.96
40%	0.84	0.94	0.92	0.78	0.96
50%	0.63	0.97	0.90	0.83	0.92
60%	0.58	0.97	0.90	0.85	0.90
70%	0.58	0.99	0.91	0.92	0.90

PPV, positive predictive value; NPV, Negative predictive value.

**Figure 5:**
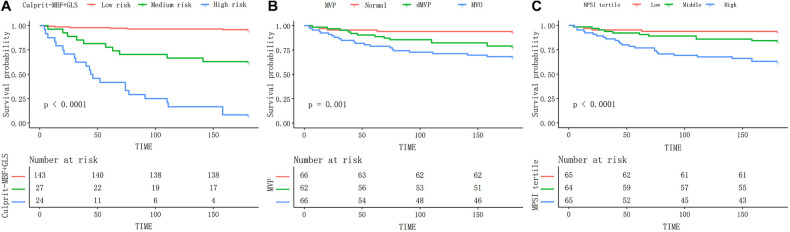
The Kaplan–Meier curves of different models during 6-month follow-up. The proposed risk stratification (**A**) exhibits better discrimination than the MVP (**B**) and MPSI (**C**) models. MBF, microvascular blood flow; MVP, microvascular perfusion; MPSI, myocardial perfusion score index.

We further stratified patients' probability of MACE at 6-month into low (≤30%), medium (>30%, ≤70%), and high risk (>70%) for better use. Distribution of MACE by different levels of risk was shown in Table 4. The event rate in high-risk patients was 91.7% compared to 31.8% for MVO and 36.9% for the high-MPSI group (Supplementary Table S3). The Kaplan-Meier curve (Figure 5) demonstrates that our proposed model can better stratify patients by proposed model than the MVP and MPSI models.

**Table 4:** Distribution of MACE in different levels of risk at 6-month follow-up.

	Low risk (≤30%)	Middle risk (>30%, ≤70%)	High risk (>70%)	*P*-value
N	143	27	24	
Cardiac death	0 (0.00%)	0 (0.00%)	5 (20.83%)	<0.01
Congestive HF	1 (0.70%)	4 (14.81%)	7 (29.17%)	<0.01
Reinfarction	2 (1.40%)	3 (11.11%)	0 (0.00%)	<0.01
Stroke	2 (1.40%)	1 (3.70%)	4 (16.67%)	<0.01
Recurrent angina	1 (0.70%)	2 (7.41%)	6 (25.00%)	<0.01
Total	6/143 (4.20%)	10/27 (37.00%)	22/24 (91.7%)	<0.01

### 3.3. Performance of automatic quantitative analysis by DNN

In total, 7,403 frames underwent myocardial segmentation by DNN. 92.45% and 7.55% of the frames were defined as “good segmentation” and “need for correction” by an experienced echocardiographer (Supplementary Table S1). Besides, the average latency (time-cost) of myocardial segmentation is 0.099s per frame on a machine with 6 cores of Inter Core i7 CPU and an NVIDIA GeForce RTX 2060 GPU. It took 248 min to obtain perfusion parameters for all patients.

### 3.4. Intra-observer and inter-observer variability of DNN assisted quantitative analysis

For intra-observer variability, the correlation coefficients of *A*, β, and MBF were 0.997 (95% CI: 0.996–0.997), 0.998 (95% CI: 0.998–0.998), and 0.999 (95% CI: 0.999–0.999). For inter-observer variability, the correlation coefficients of *A*, β, and MBF were 0.988 (95% CI: 0.985–0.990), 0.990 (95% CI: 0.988–0.992), and 0.966 (95% CI: 0.958–0.972).

## 4. Discussion

This study proposed a prognostic risk prediction model based on GLS and DNN-derived culprit-MBF for STEMI after primary PCI. It outperformed visual qualitative analysis widely used in clinical practice. Predicted risk probabilities allow for accurate prognostic stratification.

### 4.1. The microvascular dysfunction in STEMI after primary PCI

The coronary microvascular dysfunction in STEMI results from the ischemic damage and reperfusion damage to endothelial cells, increased vascular permeability and edema, inflammatory damage, microvascular injury, intramyocardial hemorrhage, platelet adhesion and aggregation, and pericyte-mediated capillary constriction (15). Many studies have proved that TIMI 3 flow by PCI does not imply adequate recovery of myocardial blood flow (4, 16–18). Indeed, perfusion of the coronary microvasculature is not fully restored in at least half of these patients, which is associated with increased morbidity and mortality (15).

Many studies have confirmed the diagnostic value of MCE quantitative analysis for microvascular dysfunction (19–22). It has the potential to identify pathologic microvascular patterns (23). It was found that 64%–81% of STEMI after PCI had microvascular dysfunction (including 29%–42% dMVP and 35%–39% MVO) (3, 4). In this study, the incidence of dMVP and MVO were 32% and 34%, close to previous studies. When using the same MACE definition, the event-free rate of dMVP and MVO at 6-month was 92% and 79%, respectively, close to previous study (dMVP: 84% and MVO: 73%). While qualitative analysis achieves patient risk stratification, the most severe microvascular dysfunction pattern MVO, still has a considerable event-free survival rate. Better risk stratification methods are expected.

### 4.2. The association between culprit-MBF&GLS and MACE

Our study indicated that the GLS and culprit-MBF derived from DNN-assisted MCE quantitative analysis were the independent protective factors of MACE. It's an objective interpretation through quantitative analysis, which could avoid empirical bias in the clinic.

The role of MCE quantitative analysis in prognostic prediction is still being explored. In patients with known or suspected coronary artery disease, quantitative stress MCE added additional prognostic information over wall motion and qualitative myocardial perfusion analysis (24). But the MCE was performed before coronary angiography with a median time interval of 18 days, and the patients who underwent coronary revascularization were excluded. The MBF was proved to be negatively correlated with the microvascular resistance after PCI, suggesting the potential prognostic prediction value (25). Wita et al. reported that quantitative perfusion parameters by MCE had high predictive value for the development of remodeling in 6-month follow-up, but the MACE wasn't investigated (26). A study with limited population has shown that MBF was an independent predictor of poor prognosis in such patients (17). While this study not only verified the prognostic value of MBF in a larger population with a more accurate and rapid method, but also developed a risk probability prediction model based on MBF and GLS.

In STEMI after PCI, the GLS is a strong predictor of left ventricular remodeling and adverse events (27–29). Speckle tracking echocardiography could distinguish the passive and active motion of myocardium. Hence, GLS mainly reflects systolic dysfunction sensitively after myocardial infarction, especially transmural infarction, which has a high risk of adverse left ventricular remodeling, heart failure and death (30, 31).

The combination of MBF and GLS allows simultaneous evaluation of hypoperfusion and systolic dysfunction, which occurs in the early and middle phases of the myocardial ischemic cascade, respectively (32). In this study, culprit-MBF (AUC: 0.89) showed excellent sensitivity (0.94) and poor specificity (0.68), while GLS (AUC: 0.77) showed better specificity (0.76) than sensitivity (0.71). The combined model achieves an optimal balance (AUC: 0.94, sensitivity: 0.84, specificity: 0.94) at the best risk threshold of 40%. The thresholds for clinical decision-making are variable and feasible, depending on the preference for active intervention or follow-up observation. Clinical decision curves can be used to evaluate sNB at various thresholds (14). In the current era of PCI, screening for microvascular dysfunction after PCI is encouraged, so a threshold probability of 20%–40% is feasible.

We divided the patients into low-, medium-, and high-risk groups according to the risk probability of 30% and 70%. Supplementary Table S4 showed that 64.5% (40/62) of dMVP and 65.2% (43/66) of MVO were reclassified to the low-risk group. Supplementary Figure S3 illustrates that the proposed model was able to perform accurate risk stratification when LVEF was greater or less than 50%. Those advantages stem not only from our model assessing both hypoperfusion and systolic dysfunction, but also from the fact that DNN-assisted quantitative analysis can make full use of spatio-temporal information. In contrast, human visual qualitative analysis is usually a crude, empirical judgment based on general impressions.

### 4.3. Predictors of MBF in the current Era

Supplementary Table S5 illustrates that the NT-proBNP and cTnI-Ultra correlated negatively with culprit-MBF among clinical variables. Among the conventional echocardiographic variables, WMSI and GLS were predictors of MBF. In contrast, other inflammatory markers, time window to PCI, LAD infarct vessel location, LVEF < 50% and diastolic function were not associated with MBF. The relationship between microvascular dysfunction and diastolic dysfunction is of interest. Previous studies found that patients with coronary flow velocity reserve < 2 appear to have higher *E*/*e*′ (*P *= 0.06) (33). However, a later study found no association between MBF measured by MCE and diastolic function (22), consistent with our findings. LAD infarct location has been shown to be independently associated with MVO (3), consistent with the univariate regression results of this study [LAD as a reference, LCA: OR 0.23 (0.09, 0.58), RCA: OR 0.08 (0.03, 0.23)], but failed to be associated with MBF. However, LAD infarct location was not a predictor of MACE in recent studies and this study. MBF was superior to MVO in predicting prognosis, and the association between LAD infarct location and MBF deserves further exploration.

### 4.4. Advantages of DNN-assisted quantitative analysis

We used a DNN-assisted MCE quantitative analysis workflow which is methodologically superior to conventional methods, has good reproducibility, and saves time and effort.

#### 4.4.1. Methodology

Take “PQ” model of Qlab 13 (Philips) as an example. It requires creating one ROI to cover myocardium at every end-systolic frame. Nevertheless, this method is sometimes unreliable because the shape and position of the heart would not always remain the same at every end-systolic phase. In contrast, the DNN could automatically trace the myocardial contour individually for every frame. See Supplementary Video S1 for comparing the two methods.

#### 4.4.2. Time-cost

In general, using “PQ” model takes about 15–20 min per patient. It would cost 2,910–3,880 min to obtain parameters for all patients. In contrast, the DNN method would cost 248 min, which is only 6%–9% of the time of the conventional method.

#### 4.4.3. Reproducibility

The variability only occurred in frames requiring manual correction, occupying only 7.55% of frames in this real-world study. Making a few corrections instead of creating the entire ROIs leads to higher reproducibility compared with previous studies (24, 34). When using conventional commercial software (“PQ” model of Qlab 13) to measure perfusion parameters in the same sample, the intra-observer correlation coefficients for A, *β* and MBF were 0.834 (95% CI: 0.796–0.866), 0.850 (95% CI: 0.819–0.877) and 0.826 (95% CI: 0.780–0.861), respectively. Inter-observer correlation coefficients were 0.746 (95% CI: 0.685–0.794), 0.722 (95% CI: 0.665–0.770), 0.711 (95% CI: 0.634–0.771) for A, *β* and MBF, respectively.

### 4.5. Limitations

Since this study is a single-center with short-term follow-up study, the predictive models developed in this study should be externally validated in other populations or settings to determine their generalizability and clinical utility. And longer follow-up is needed to explore the long-term prognostic value of proposed model. Our proposed automatic segmentation DNN still requires manual correction in few frames. We emphasize that the purpose of artificial intelligence at this stage is to reduce the difficulty and intensity of work through human-computer interaction. Myocardial segments ascribed to left circumflex artery and right coronary artery were predetermined, which may be influenced by coronary dominance. The pre-PCI TIMI flow, thrombus burden, and echocardiographic follow-up were not available in this study but were worth exploring for future research.

## 5. Conclusion

This study employed a standardized process for DNN-assisted MCE quantitative analysis. It revealed that among STEMI with the restoration of TIMI 3 flow, culprit-MBF and GLS were independent predictors of short-term prognosis. The proposed risk prediction model allowed for accurate risk stratification and outperformed conventional visual qualitative analysis.

## Data Availability

The raw data supporting the conclusions of this article will be made available by the authors, without undue reservation.
