# Supplementary material for: A novel risk stratification model for STEMI after primary PCI: global longitudinal strain and deep neural network assisted myocardial contrast echocardiography quantitative analysis
Source: Front Cardiovasc Med. 2023 Apr 27;10:1140025. doi: 10.3389/fcvm.2023.1140025 (PMC10172492; doi:10.3389/fcvm.2023.1140025)
Supplement: Supplementary file 2 [file Table1.docx]

Supplementary Material

A Novel Risk Stratification Model for STEMI after Primary PCI: Global Longitudinal Strain and Deep Neural Network Assisted Myocardial Contrast Echocardiography Quantitative Analysis

Mingqi Li†, Dewen Zeng†, Yanxiang Zhou, Jinling Chen, Sheng Cao, Hongning Song, Bo Hu, Wenyue Yuan, Jing Chen, Yuanting Yang, Hao Wang, Hongwen Fei, Yiyu Shi, Qing Zhou*

†These authors contributed equally to this work and share first authorship

*** Correspondence:** Qing Zhou: qingzhou.wh.edu@hotmail.com

# Supplementary Tables

**Supplementary Table 1. Performance of** **automatic segmentation by the deep neural network.**

|  | N (frames) | Good segmentation | Need for correction |
| --- | --- | --- | --- |
| A4C | 2481 | 2307 (92.99%) | 174 (7.01%) |
| A2C | 2446 | 2252 (92.07%) | 194 (7.93%) |
| A3C | 2476 | 2285 (92.29%) | 191 (7.71%) |
| Total | 7403 | 6844 (92.45%) | 559 (7.55%) |

A2C apical two-chamber; A3C apical three-chamber; A4C: apical four-chamber

**Supplementary Table 2. Prognostic prediction performance of proposed model at1-, 3-, 6-, 9- and 12-month follow-up.**

|  | AUC | Best threshold | Sensitivity | Specificity |
| --- | --- | --- | --- | --- |
| 1m | 0.88 (0.79-0.94) | 12% | 0.81 | 0.85 |
| 3m | 0.92 (0.87-0.96) | 30% | 0.84 | 0.91 |
| 6m | 0.94 (0.91-0.98) | 40% | 0.84 | 0.94 |
| 9m | 0.94 (0.90-0.97) | 43% | 0.83 | 0.95 |
| 12m | 0.94 (0.90-0.97) | 29% | 0.86 | 0.88 |

**Supplementary Table 3. Distribution of MACE in MVP qualitative analysis at 6-month follow-up**

|  | MVP | | |  | MPSI | | |  |
| --- | --- | --- | --- | --- | --- | --- | --- | --- |
|  | Normal | dMVP | MVO | *P*-value | Low | Middle | High | *P*-value |
| N | 66 | 62 | 66 |  | 65 | 64 | 65 |  |
| Cardiac death | 0 (0.00%) | 0 (0.00%) | 5 (7.58%) | < 0.01 | 0 (0.00%) | 0 (0.00%) | 5 (7.58%) | < 0.01 |
| Congestive HF | 2 (3.03%) | 3 (4.84%) | 7 (10.61%) | 0.17 | 2 (3.08) | 0 (0.00%) | 10 (15.38) | < 0.01 |
| Reinfarction | 0 (0.70%) | 3 (4.84%) | 2 (3.03%) | 0.22 | 0 (0.00%) | 4 (6.25%) | 1 (1.54%) | 0.07 |
| Stroke | 2 (3.03%) | 2 (3.23%) | 3 (4.55%) | 0.88 | 2 (3.08) | 1 (1.56%) | 4 (6.15%) | 0.36 |
| Recurrent angina | 0 (0.00%) | 5 (8.06%) | 4 (6.06%) | 0.08 | 0 (0.00%) | 5 (7.81%) | 4 (6.15%) | 0.08 |
| Total | 4 (6.06%) | 13 (20.97%) | 21 (31.82%) | < 0.01 | 4 (6.15%) | 10 (15.62%) | 24 (36.92%) | < 0.01 |

dMVP: delayed microvascular perfusion; MPSI: myocardial perfusion score index; MVO: microvascular obstruction.

**Supplementary Table 4. Distribution of three patterns of MVP in different risks level**

|  | Normal MVP | dMVP | MVO | *P*-value |
| --- | --- | --- | --- | --- |
| Predicted risk |  |  |  | <0.001 |
| ≤ 0.3 | 60 (90.91%) | 40 (64.52%) | 43 (65.15%) |  |
| > 0.3, ≤ 0.7 | 5 (7.58%) | 13 (20.97%) | 9 (13.64%) |  |
| > 0.7 | 1 (1.52%) | 9 (14.52%) | 14 (21.21%) |  |

dMVP: delayed microvascular perfusion; MVO: microvascular obstruction.

**Supplementary Table 5. Predictor of culprit-MBF by univariate regression analysis**

|  | β (95% CI) | *P*-value |
| --- | --- | --- |
| Male | 8.20 (-5.58, 21.99) | 0.25 |
| Age | 0.26 (-0.20, 0.72) | 0.26 |
| Killip |  |  |
| I | Ref |  |
| II | 2.41 (-11.50, 16.31) | 0.74 |
| III | -14.43 (-31.61, 2.75) | 0.10 |
| IV | -9.98 (-24.00, 4.04) | 0.16 |
| Hypertension | -8.26 (-19.21, 2.70) | 0.14 |
| Diabetes mellitus | -3.73 (-15.46, 8.01) | 0.53 |
| Time window to PCI | -0.22 (-0.54, 0.10) | 0.18 |
| Systolic blood pressure (mmHg) | -0.10 (-0.37, 0.17) | 0.46 |
| NT-proBNP (ng/ml) per 100 increases | -0.21 (-0.35, -0.07) | **< 0.01** |
| Leukocyte (10^9^/L) | -0.94 (-2.53, 0.66) | 0.25 |
| Hs-CRP (mg/L) | -0.08 (-0.26, 0.11) | 0.41 |
| Creatinine (umol/L) | 0.01 (-0.20, 0.21) | 0.96 |
| CK-MB (ng/mL) | -0.01 (-0.06, 0.04) | 0.70 |
| Myoglobin (ug/L) | 0.00 (-0.01, 0.02) | 0.63 |
| cTnI-Ultra (ng/mL) | -0.45 (-0.76, -0.14) | **< 0.01** |
| Culprit vessel |  |  |
| LAD | Ref |  |
| LCx | -4.97 (-19.73, 9.78) | 0.51 |
| RCA | 8.23 (-4.02, 20.49) | 0.19 |
| Number of stenosed vessels |  |  |
| 1 | Ref |  |
| 2 | -1.12 (-13.16, 10.92) | 0.86 |
| 3 | -12.62 (-34.43, 9.20) | 0.26 |
| LVEF (%) | 0.60 (0.00, 1.19) | **0.05** |
| < 50% | -5.27 (-16.04, 5.49) | 0.34 |
| LVEDV (ml) | 0.03 (-0.13, 0.20) | 0.67 |
| LAV Index (ml/m^2^) | 0.11 (-0.43, 0.64) | 0.69 |
| E/A | 8.02 (-6.59, 22.62) | 0.28 |
| E/e’ | 0.37 (-1.12, 1.85) | 0.63 |
| TAPSE (mm) | -0.52 (-2.83, 1.80) | 0.66 |
| GLS (%) | 2.41 (1.16, 3.66) | **< 0.01** |
| WMSI per 0.1 increase | -2.24 (-3.94, -0.54) | **0.01** |

CK-MB: creatine kinase myocardial band; cTnI: cardiac troponin I; E/A: The ratio of peak mitral valve velocity of early (E) and late (A) diastole. E/e’: The ratio of E and myocardial peak early velocity at medial mitral annulus. GLS: global longitudinal strain; Hs-CRP: high-sensitivity C-reactive protein; LAV-index: Left atrial volume divided by body surface area; LVEDV: left ventricular end-diastolic volume; LVEF: left ventricular ejection fraction; NT-proBNP: N-terminal pro b-type natriuretic peptide; PCI: percutaneous coronary intervention; TAPSE: tricuspid annular plane systolic excursion; WMSI: wall motion score index

# Supplementary Figures


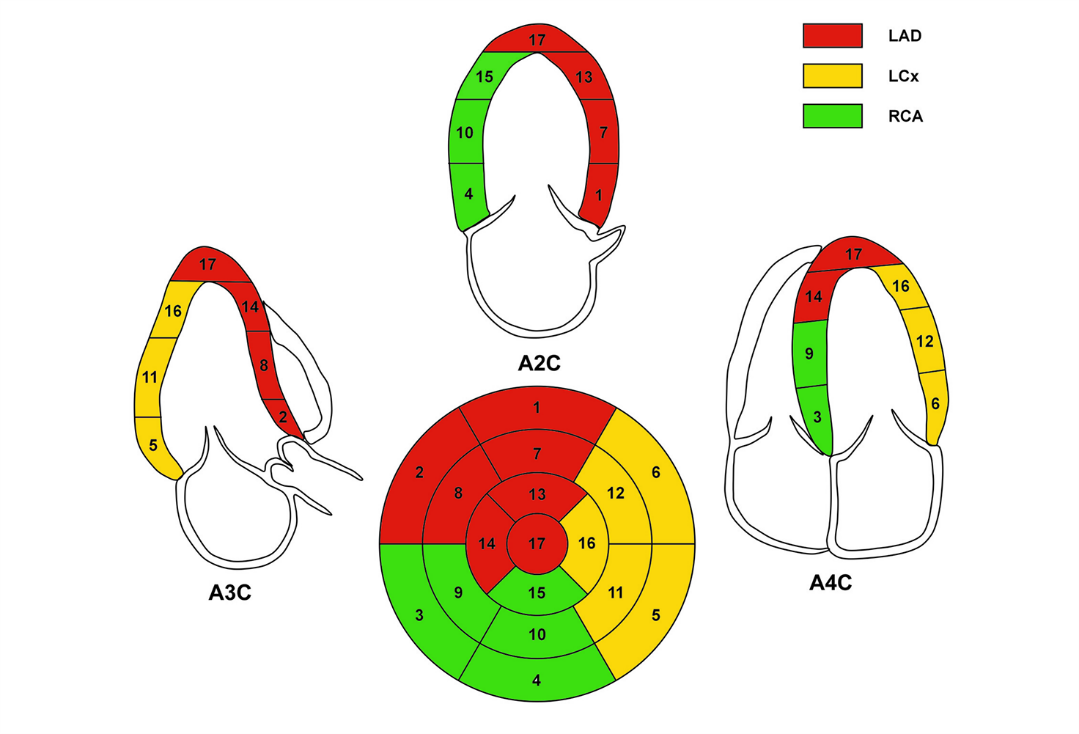


**Supplementary Figure 1.** Standardized 17-segment of the myocardium and coronary territories. LAD: left anterior descending artery; LCx: left circumflex artery; RCA: right coronary artery.


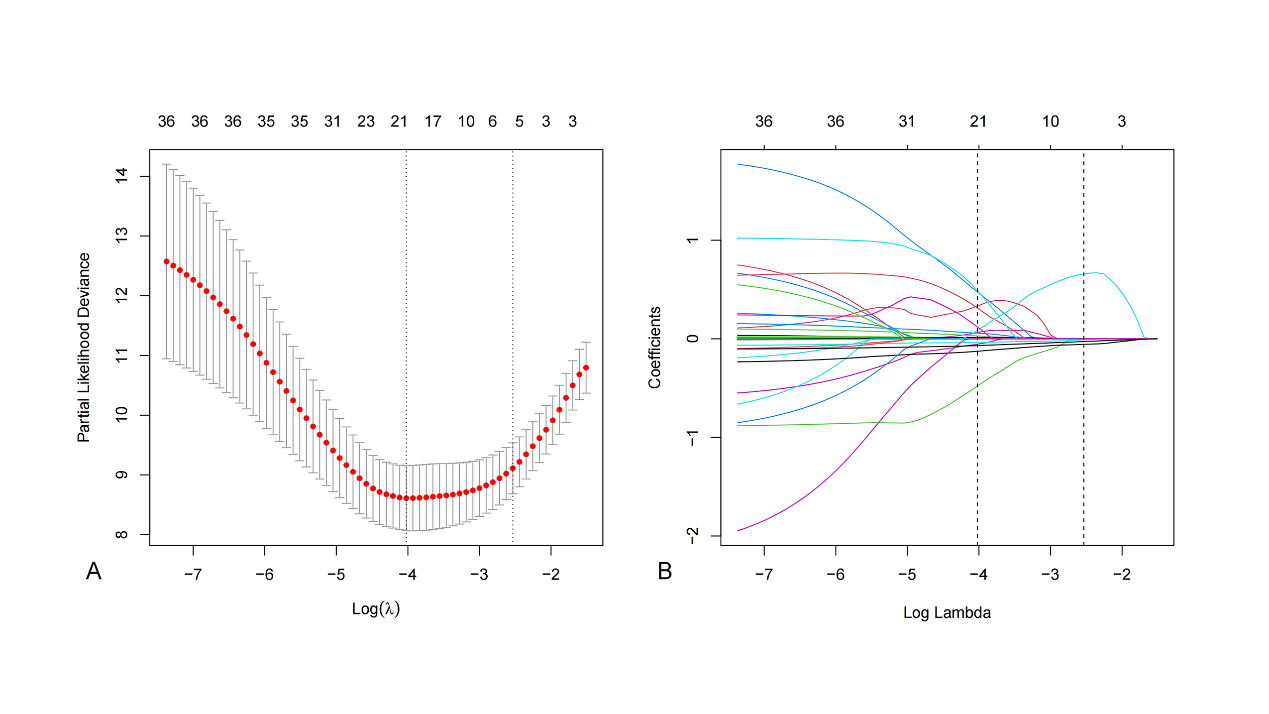


**Supplementary Figure 2.** LASSO logistic regression for initial variable selection. 29 variables were included for LASSO regression analysis (Age, gender, hypertension, diabetes mellitus, admission glucose, Killip level, time window to PCI, systolic blood pressure, NT-proBNP, leukocyte, Hs-CRP, creatinine, CK-MB, myoglobin, cTnI-Ultra, LVEF, LVEDD, LAV-index, E/A, E/e’, TAPSE, MVP, WMSI, MPSI, GLS, number of stenosed vessels, culprit vessel, culprit-β, culprit-MBF). **(A)** Tuning parameter (lambda: λ) selection in the LASSO model used 10-fold cross-validation via minimum criteria; **(B)** LASSO coefficient profiles of the features against the log(λ).


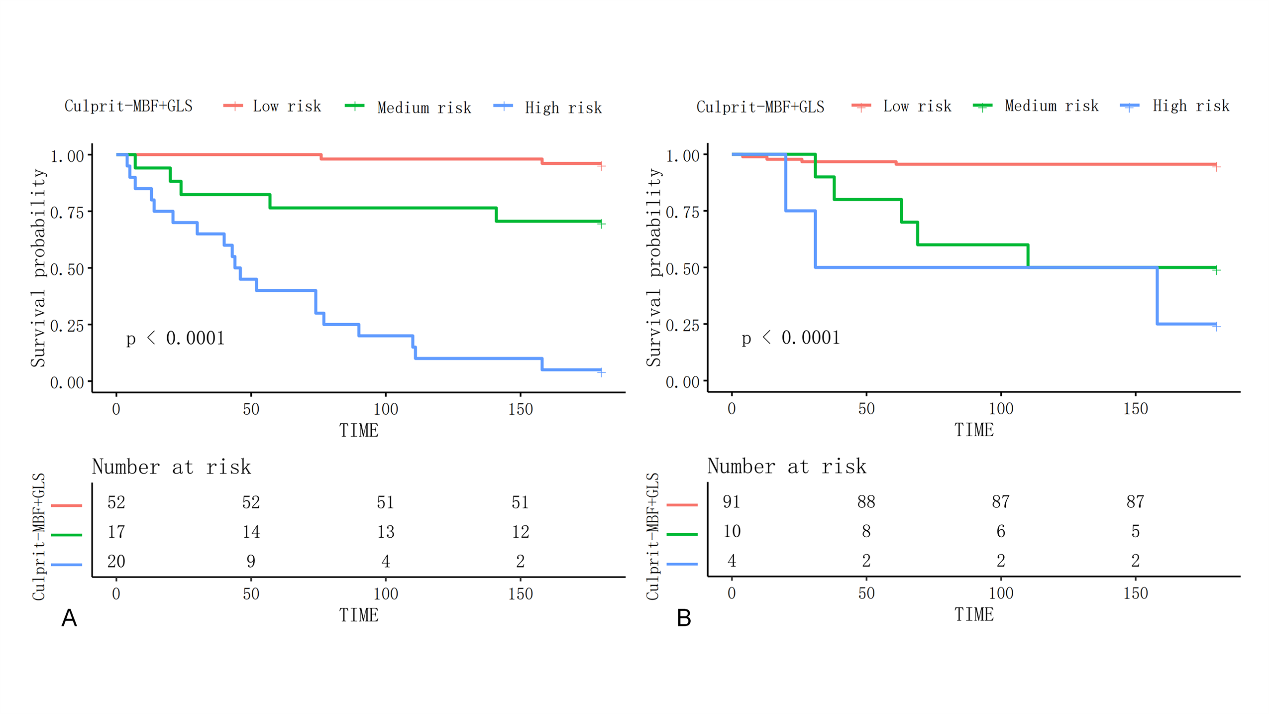


**Supplementary Figure 3.** The Kaplan-Meier curves of proposed risk model among LVEF less **(A)** or greater than 50% **(B)**.

**Supplementary Video 1.** Comparison of DNN method and manual method by commercial software for MCE myocardial segmentation. The left is the input raw MCE image. The middle is the DNN-predicted myocardial mask (below) and the ROI placed manually based on the first systolic frame (up), which is assigned to cover all myocardium of the rest frames. The red arrows point to segmentation errors; The right is the final output of the DNN-segmented myocardium (below) and the failed-fit area (blue) by commercial software (up). S1-n: end-systolic frames of consecutive cardiac cycles.
